# Role of Point Defects and Ion Intercalation in Two-Dimensional Multilayer Transition Metal Dichalcogenide Memristors

**DOI:** 10.1021/acsanm.4c04769

**Published:** 2024-10-24

**Authors:** Mohit D. Ganeriwala, Alejandro Toral-López, Estela Calaforra-Ayuso, Francisco Pasadas, Francisco G. Ruiz, Enrique G. Marin, Andres Godoy

**Affiliations:** Department of Electronics and Computer Technology, University of Granada, 18071 Granada, Spain

**Keywords:** neuromorphic computing, synapse, memristor, 2D materials, transition metal dichalcogenides, sulfur vacancy, metal intercalation

## Abstract

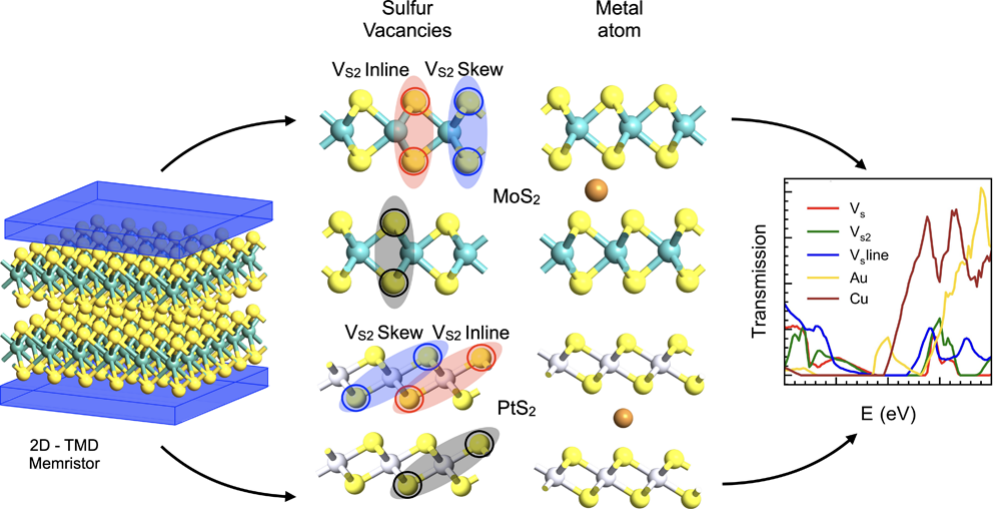

Two-dimensional materials, in particular transition metal dichalcogenides (TMDs), have attracted a nascent interest in the implementation of memristive architectures. In addition to being functionally similar to synapses, their nanoscale footprint promises to achieve the high density of a biological neural network in the context of neuromorphic computing. However, in order to advance from the current exploratory phase and reach reliable and sound memristive performances, an understanding of the underlying physical mechanisms in TMD memristors seems imperative. Despite the distinctive transport medium inherent to multilayer TMDs, the memristance is routinely attributed to defects or metal atoms present in the system, with their precise contribution remaining elusive. Specifically, the role of intrinsic point defects in the formation of conductive channels, although shown for monolayer TMDs, is not conclusively studied for multilayer samples. In this work, using density functional theory (DFT) and nonequilibrium Green’s function (NEGF) formalism, a systematic study is carried out to analyze the impact that defects and metal atoms produce on the out-of-plane conductivity of multilayer TMDs. MoS_2_, a representative of the 2H structural configuration, and PtS_2_, a representative of the 1T structure, the most common crystal arrangements among TMDs, are used for this analysis. It is found that the intrinsic sulfur vacancies, which are the dominant defects present in both TMDs, appear to be insufficient in causing resistive switching on the application of an external bias. The claim that the intrinsic point defects on their own can realize a valence change memory-type device by providing a controllable conductive channel through the van der Waals structure seems, according to our study, improbable. The presence of metallic atoms is demonstrated to be essential to trigger the memristive mechanism, emphasizing the proper choice of a metal electrode as being critical in the fabrication and optimization of memristors using TMDs.

## Introduction

Resistive switching devices, also known as memristors, have shown the potential to closely mimic the functionality of biological synapses and are, thus, expected to become a keystone for future human brain-inspired neuromorphic systems.^1,2^ While resistive switching has already been investigated for more than half a century, mostly making use of metal–insulator–metal (MIM) structures based on transition metal oxides (TMOs),^3−5^ the advent of two-dimensional materials (2DMs) has given a twist to the field. In particular, 2DM-based memristors are expected to boost essential aspects for the deployment of bioinspired systems such as heterointegration, flexibility, low power operation, and high integration density.^6−8^ Several 2DMs such as graphene, transition metal dichalcogenides (TMDs), and hexagonal boron nitride (h-BN), as well as their combinations, have already been reported to show resistive switching behavior.^9−11^ However, despite the promises already shown, memristors based on 2DMs are still in their infancy in terms of their technological advancement, and their performance needs to be improved to reach the maturity of the state-of-the-art TMO realizations.^9,10^ To overcome this exploratory phase, it becomes crucial, in addition to continuous experimental efforts, to develop a comprehensive understanding of the physical processes that control and modulate the switching phenomena in 2DMs.

In order to guide this exploration in the 2D memristive flatland, it is useful to look at the physical mechanisms involved in the modification of the conductivity of insulators in the already charted conventional TMO-based architectures. In these devices, the most common resistive switching mechanism implies some kind of ionic motion through the insulator, usually classified into two main types:^3^ (i) valence change memory (VCM), where the vacancies of one of the constituent elements of the insulator govern the switching, and (ii) electrochemical metallization memory (ECM), where atoms of the electrode material play the leading role in the process. In more detail, in VCM-type devices, the rearrangement of oxygen vacancies inside the TMOs alters the valence state of the transition metal cation, forming a virtual filament that induces the change in the conductivity of the structure. On the other hand, in ECM-type devices, the metal atoms introduced by the electrode form a metallic filament of high conductivity. The formation and rupture of both the virtual and metallic filaments can be controlled by an externally applied bias.

This classification is frequently employed to explain the experimental results achieved in 2DM-based memristors. For example, both virtual filaments due to vacancies and physical filaments generated by metal atoms are argued to be responsible for resistive switching in insulating h-BN-based memristors.^12,13^ In addition to h-BN, several semiconducting TMDs have also demonstrated resistive switching capabilities, e.g., MoS_2_-based devices, where active metal atoms such as Cu and Ag are assumed to be the origin of the memristive effect.^14,15^ Other TMDs, such as MoSe_2_, MoTe_2_, PtSe_2_, and WSe_2_, both monolayer and few-layer thick, have also shown resistive switching capabilities with the inert metal Au used to form the electrodes.^16^ Some studies have attributed the resistive switching in these structures to the migration of sulfur vacancies, possibly drift by the electric field and/or temperature gradients forming filaments, while others claim that the switching is due to the interplay between sulfur vacancies and Au.^17−20^ Resistive changes in response to an external field in multilayer WS_2_-based devices have been also ascribed to modifications in the tunneling barrier caused by the redistribution of sulfur vacancies.^21^ Monolayer MoS_2_ with in-plane transport has also shown memristive switching attributed to the presence of grain boundaries.^22^

The resistive switching mechanism in 2DM-based memristors is therefore attributed to the defects or metal atoms present in the system, with their precise contribution remaining elusive. Exploration at the atomistic level could provide valuable insights into the role of the constituent species in the observed memristance. While such studies have proved valuable in the exploration of the physical mechanism of bulk oxide-based memristors,^23^ similar studies in 2D TMDs are often limited to monolayers.^16,20^ However, in 2DM vertical stacks, where electron transport is along the out-of-plane direction, the transport medium is rather different. Thus, while the presence of vacancies and other defects has shown to increase the in-plane conductivity, the out-of-plane conductivity might instead be limited by the tunneling barrier originated at the interlayer van der Waals (vdW) gaps.^19,24,25^ The claim that the intrinsic point defects in TMDs are paramount for realizing VCM-type devices by forming the conductive channel through the vdW gaps has not been extensively analyzed.^17^ Recently, a theoretical study^13^ has shown how the clustering of defects in h-BN layers modifies the vdW gaps by forming physical bonds (interlayer bridges) and the control exerted on them by an external bias. This pioneering work is restricted to h-BN, as it depends on the specific defect configuration found in this material, not common among other 2DMs and specifically in TMDs, where further exploration of the impact of this effect is needed. In particular, as a large number of semiconducting TMDs are experimentally assessed for their memristive effect, it is important to evaluate the sufficiency of the intrinsic defects found in TMDs to originate a modification of the conductivity in response to an external bias or if the choice of the appropriate metal electrode is imperative in realizing TMD-based memristors.

To gain further insights into these questions, this work makes use of density functional theory (DFT) and the nonequilibrium Green’s function (NEGF) formalism to carry out a systematic study of the role played by the intrinsic point defects and metal atoms in the modification of the out-of-plane conductivity of two chosen TMDs: MoS_2_, as a representative of the 2H structural configuration, and PtS_2_, a representative of the 1T structure.

## Computational Details

### DFT Simulations

DFT calculations are carried out using the generalized gradient approximation (GGA) as implemented in QuantumATK with the linear combination of atomic orbitals (LCAO) method using the Perdew–Berke–Erzenhof exchange-correlation functional.^26^ The LCAO describes the electronic structure using norm-conserving pseudopotentials with considerable accuracy and lower computational time than the projector augmented wave (PAW) method. This allows the consideration of larger supercells as well as bilayer structures. The SG15 pseudopotential with a medium-size basis set is considered for the MoS_2_ simulations, while the OpenMX pseudopotential with spin–orbital coupling is considered for the PtS_2_ simulations. Brillouin zone integration was performed over a grid of *k*-points with a density (Å) of 4 × 4 × 1 for the mono- and bilayer and 4 × 4 × 4 for the bulk in the Monkhorst–Pack scheme. A sufficiently large energy cutoff of 250 Ry was taken. To avoid any interaction between the periodic images of the neighboring slabs in mono- and bilayer structures, a vacuum region of at least 20 Å is included in the out-of-plane direction. The van der Waals (vdW) interactions between layers in the bilayer and bulk structures are taken into account in the calculations through Grimme’s DFT-D2 dispersion corrections. The geometries and lattice parameters were fully relaxed until the forces acting on each atom were <0.01 eV/Å. The validation of the DFT simulations was established through comparison with the electronic band structure of the monolayer, bilayer, and bulk MoS_2_ and PtS_2_ reported in the literature,^27,28^ as shown in the Supporting Information (S.I.), Figure S1.

### Calculation of the Formation Energy

The formation energy per defect/external metal atom is calculated using the following expression^29^
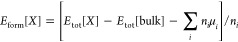
1where *E*_tot_[*X*] is the total energy of the host crystal including defects or external metal atoms, *E*_tot_[bulk] is the total energy of the pristine crystal without defects, and μ_*i*_ is the chemical potential of the *n*_*i*_ added or subtracted atoms of element *i*. To calculate the chemical potential, the metal (Mo/Pt) and sulfur (S) are considered to be in thermal equilibrium with the respective materials (MoS_2_ and PtS_2_).^30^ It has been shown both experimentally and theoretically that the sulfur vacancy is the predominate point defects in both MoS_2_ and PtS_2_.^29,31^ The same remark is confirmed here through simulation (S.I., Figure S2). It can be seen that for the μ_s_ ranging from Mo/Pt-rich to sulfur-rich, the sulfur vacancy defect shows *E*_form_ lower than the Mo/Pt vacancy defect. Note also from S.I. Figure S2 that the choice of chemical potential affects only the absolute value of *E*_form_. Thus, the relative energies and, therefore, the conclusions drawn are independent of the value of chemical potential, provided that a consistent value is used throughout the study. Therefore, all the calculations reported in this work are performed by considering the sulfur chemical potential μ_s_ for the sulfur-rich condition of MoS_2_ or PtS_2_.

### Quantum Transport Simulation

The quantum transport simulations are carried out using the fully self-consistent DFT–NEGF formalism. The optimization and *k*-point density of the DFT are kept the same as used for the electronic structure calculations. The vdW forces are also considered here using Grimm’s DFT-D2 corrections. Due to the huge computational burden of this study, only the zero-bias scenario is analyzed. The transport peak at and around the Fermi level can thus give an idea of the zero-bias conductance of the system. The device structure is modeled using a bulk 3 × 3 supercell with the previously found stable configuration of the vacancy defects and intercalated metallic atoms. Therefore, the central region and electrode have the same composition.

## Results

### Structural and Electronic Properties

#### Intrinsic Defects

The validity of the proposed theoretical framework is first accessed by studying the dominant defect in TMDs, which is known to be the single neutral chalcogen vacancy.^30−32^ To confirm that the simulation setup produces results consistent with the literature, different monolayer MoS_2_ and PtS_2_ supercells, ranging from 6 × 6 down to 2 × 2 (see S.I. Figure S3), with a single sulfur vacancy (*V*_S_) are investigated (see also S.I. Table S1 for the corresponding values of the formation energies). The formation energy (*E*_form_) remains constant until the supercell size is reduced to 3 × 3, beyond which *E*_form_ increases, pointing out that the 3 × 3 supercell (with a single *V*_S_) is the minimum size preventing vacancy interactions. The single *V*_S_ in a 3 × 3 supercell corresponds to a concentration of ∼10^14^ cm^–2^ for MoS_2_ and ∼8 × 10^13^ cm^–2^ for PtS_2_, while the single *V*_S_ in 4 × 4, 5 × 5, and 6 × 6 correlates with concentrations in the range of ∼6 × 10^13^ to ∼3 × 10^13^ cm^–2^ for MoS_2_ and ∼5 × 10^13^ and ∼2 × 10^13^ cm^–2^ for PtS_2_. These values are in good agreement with CVD-grown samples of TMDs and other theoretical studies, where the *V*_S_ is measured to reach a concentration of around 10^13^ cm^–2^.^33,34^ The formation energy (*E*_form_) for *V*_S_ is found to be 2.75 and 1.79 eV for MoS_2_ and PtS_2_, respectively, which is 1.41 and 1.57 eV lower than the single transition metal vacancy (further details on *V*_Mo_ or *V*_Pt_ in Figure S2 of the S.I.), evidencing that the *V*_S_ is energetically more likely to be present in CVD-grown MoS_2_ and PtS_2_, in very good agreement with the literature.^30,31,34^

**Figure 1:**
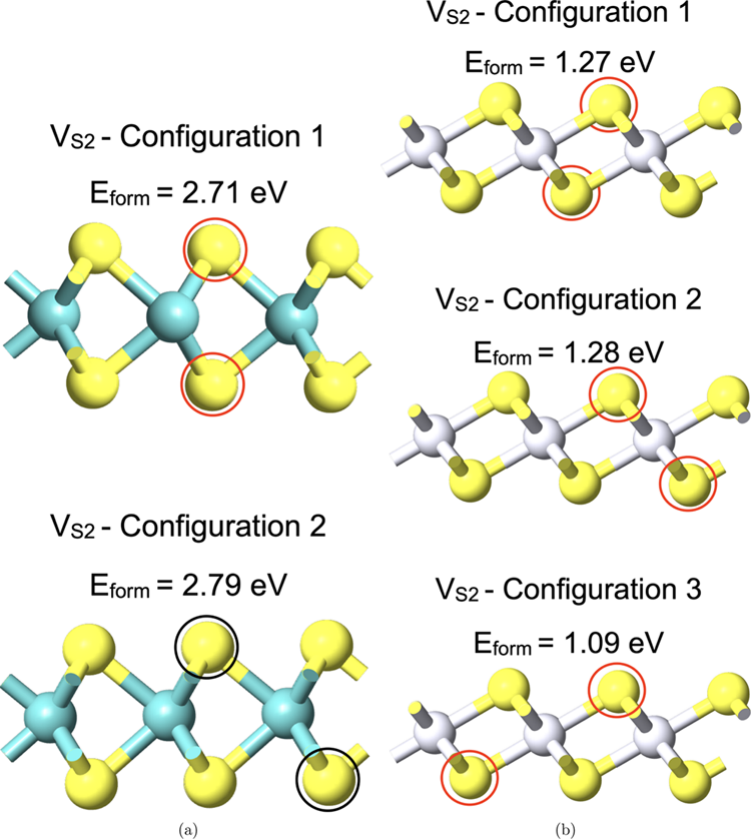
Side view of the supercell with the sulfur divacancy *V*_S2_ point defect and the corresponding energy of formation, *E*_form_: (a) two configurations for MoS_2_ and (b) three configurations for PtS_2_.

Next, divacancies (*V*_S2_) placed on opposite sulfur planes are studied. Two possible configurations of *V*_S2_ for MoS_2_ and three for PtS_2_ (Figure 1) are analyzed, and the calculated *E*_form_ is mentioned alongside in Figure 1. It can be seen that Configuration 1 (up–down) for MoS_2_ and Configuration 3 (staggered) for PtS_2_ exhibit the lowest *E*_form_.

**Figure 2:**
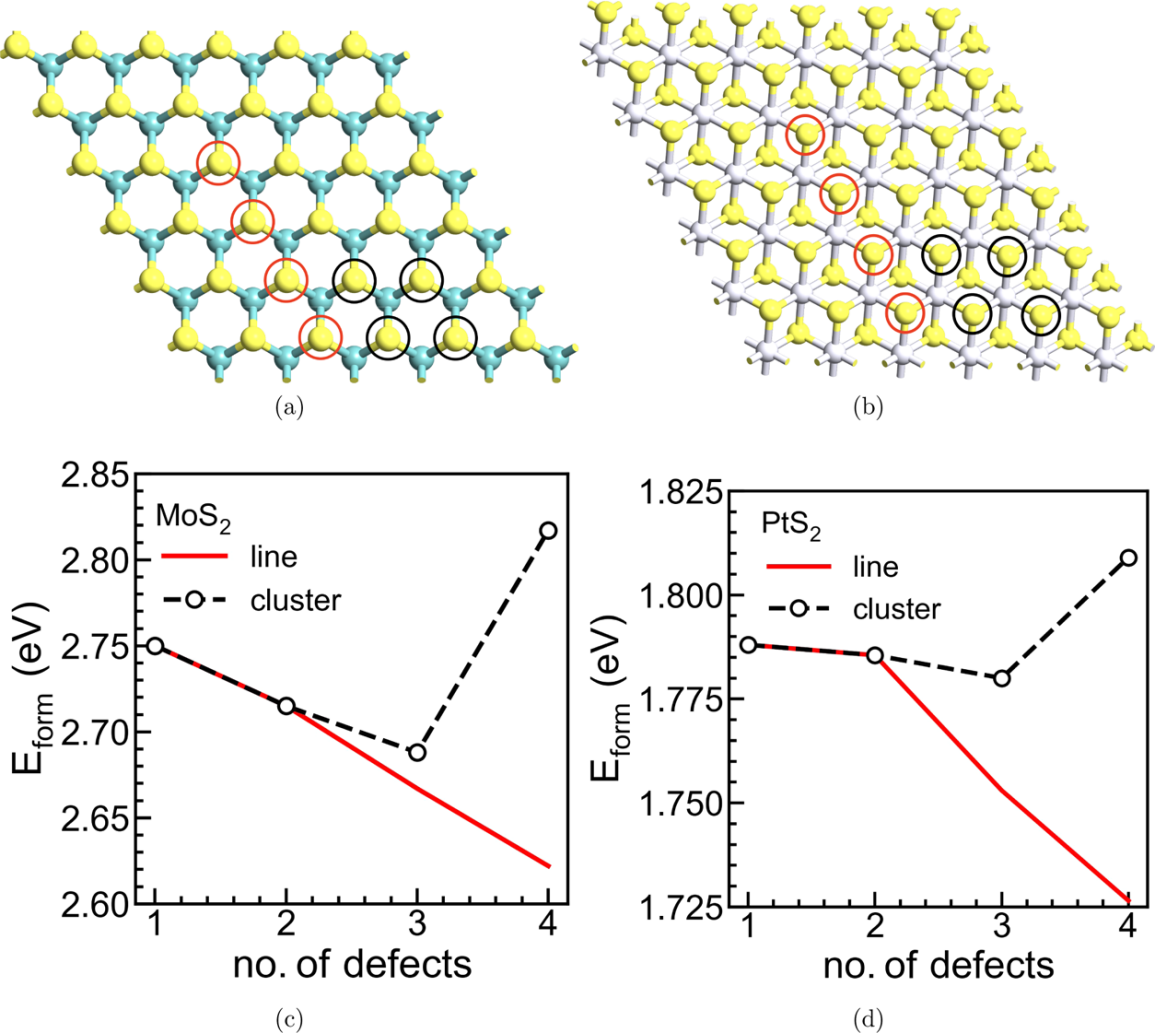
Top view of a 6 × 6 supercell with the single sulfur vacancy *V*_s_ forming the extended line and localized cluster for (a) MoS_2_ and (b) PtS_2_. The variation in formation energy (*E*_form_) per *V*_s_ for line defect vs cluster for (c) MoS_2_ and (d) PtS_2_.

As the concentration of sulfur vacancies increases, they can either be randomly distributed or start interacting to form an extended defect configuration. Thus, the clustering of vacancies could give rise to localized states, which can lead to an increased level of localized transport in vertical structures. Such clustering has, indeed, shown to be associated with virtual filament formation in the case of h-BN.^13^ Extended line defects versus localized clustering of the *V*_S_ are next compared based on the *E*_form_ evolution. Figure 2a,2b shows the MoS_2_ and PtS_2_ structure, respectively, with the extended line (S atoms circled in red) and localized (S atoms circled in black) *V*_S_ clustering. Either the line or the cluster arrangements are simulated separately. Figure 2c,2d displays the variation of *E*_form_ per sulfur vacancy, i.e., as a new *V*_S_ is added to the previously existing configuration. As shown, in the case of the line defect, there is a reduction in the *E*_form_ per *V*_S_ with each new *V*_S_. On the contrary, for the cluster arrangement, the *E*_form_ evolution per *V*_S_ is not monotonic. When the number of *V*_S_ is higher than 3 (where the cluster actually distinguishes itself from a line), the *E*_form_ per *V*_S_ increases, proving that it is energetically less stable to form a localized cluster in MoS_2_ and PtS_2_. Note that the two simplified scenarios of *V*_S_ clustering considered here, although not exhaustive, agree with the main conclusions claimed in the literature, i.e., the preferential formation of *V*_S_ extended lines in MoS_2_.^29,35^ Such extended defect structures are shown to increase the in-plane conductivity;^24^ however, their effect on the out-of-plane transport has not been already analyzed in TMDs.

**Figure 3:**
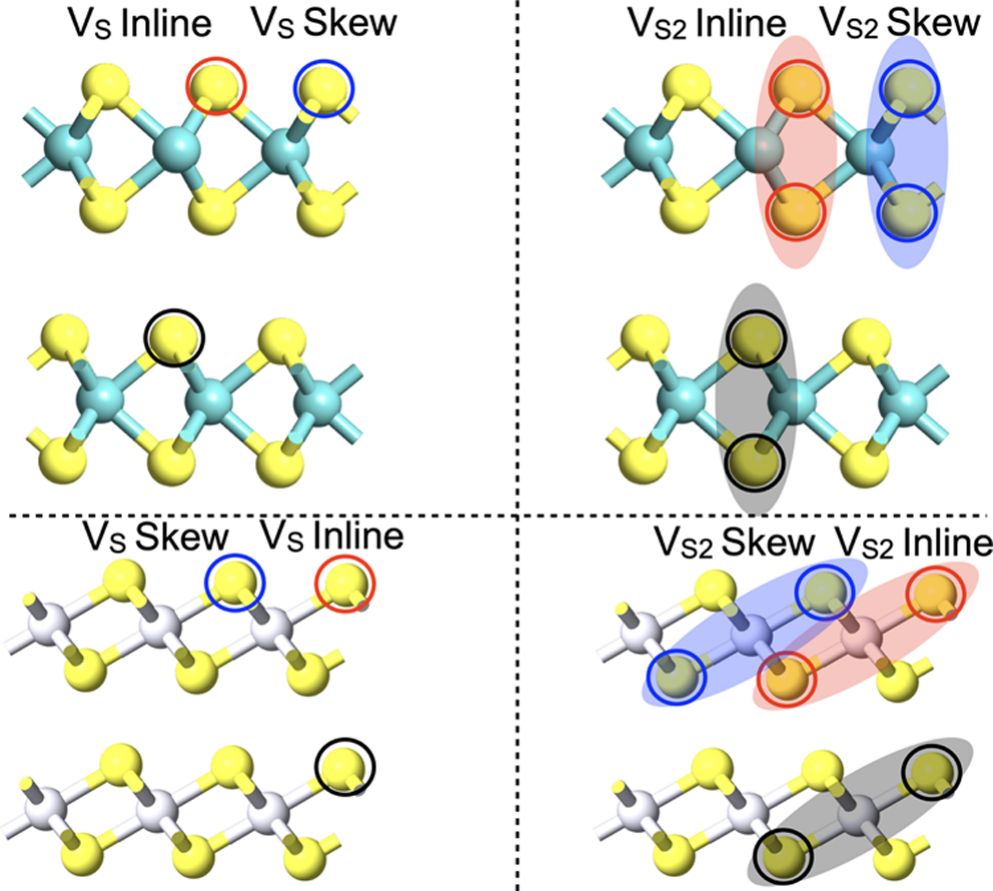
Side view of a 3 × 3 supercell for bilayer MoS_2_ (top row) and PtS_2_ (bottom row) with single vacancy (*V*_S_) or divacancy (*V*_S2_) sulfur defects. Given the vacancy placement in a layer (circled in black), there are two different possibilities for the alignment of the vacancy in the adjacent layer: vertically aligned (circled red) and vertically skewed (circled blue).

**Figure 4:**
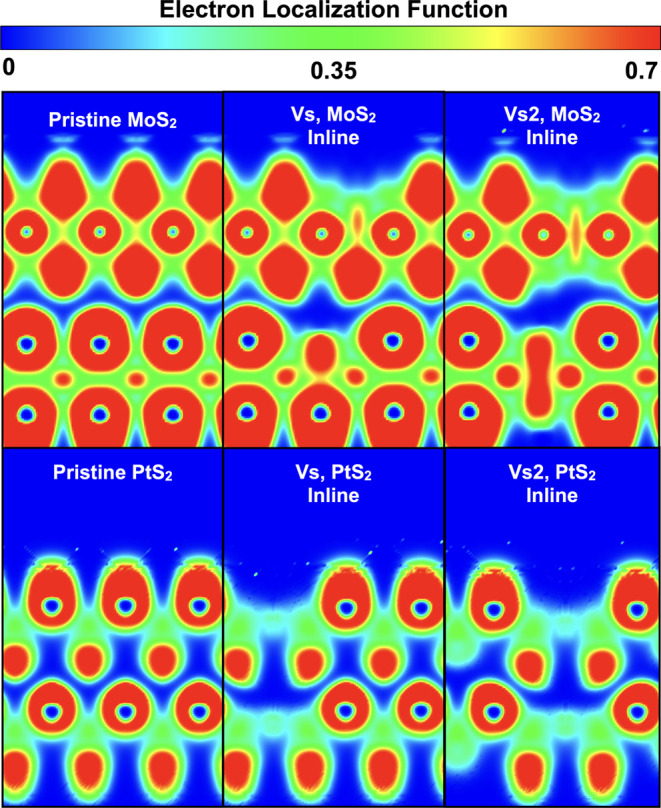
Electron localization function (ELF) for MoS_2_ (top row) and PtS_2_ (bottom row) for pristine, single sulfur point defects (*V*_S_) and sulfur divacancy defects (*V*_S2_).

To advance the understanding of the vertical memristive structure, the arrangement of various sulfur vacancies in a bilayer and their impact on the vdW gap are then examined. Figure 3 presents the 3 × 3 × 2 bilayer structure, with both *V*_S_- and *V*_S2_-type sulfur vacancies. Given the vacancy placement in a layer (circled in black), there are two different possibilities of the vacancy alignment in the adjacent layer, viz., (a) vertically aligned (circled red) and (b) vertically skewed (circled blue), as shown in Figure 3. Note that the 3 × 3 × 2 supercell size is chosen to maintain the highest concentration of vacancies. The calculated *E*_form_ for both structures is gathered in Table 1. The data obtained indicate that the position of a vacancy in one of the layers does not produce a noticeable influence on the position (and also the type, see S.I. Table S2) of the other vacancy in the adjacent layer, revealing the low interaction between both layers. Focusing further on the vertically aligned vacancy structure, the bilayer distance *d*_S–S_ of the pristine MoS_2_, which is found to be 3.27 Å, is reduced to 3.01 Å for both the *V*_S_- and *V*_S2_-type vacancies, while for PtS_2_, the pristine *d*_S–S_ of 2.61 Å reduces to 2.42 and 2.38 Å for *V*_S_ and *V*_S2_, respectively. The reduction of bilayer distance suggests a reduction in the tunneling gap that could lead to the formation of bonds, increasing the out-of-plane electron transmission and conductance. To further analyze this scenario, the electron localization function (ELF) is evaluated for the different sulfur vacancy configurations (Figure 4). As can be observed, while the presence of sulfur vacancies reduces the tunneling barrier for the out-of-plane electron transport by approximately 7–9%, it does not introduce any apparent physical bonds between layers.

**Table 1:** Formation Energy (*E*_form_) and Bilayer Distance (*d*_s-s_) for MoS_2_ and PtS_2_ for Various Bilayer Defect Configurations

		MoS_2_		PtS_2_	
defect	structure	*E*_form_ (eV)	*d*_s-s_ (Å)	*E*_form_ (eV)	*d*_s-s_ (Å)
**pristine**			3.27		2.61
***V***_**s**_	**inline**	2.98	3.01	1.91	2.42
	**skew**	2.98		1.91	
***V***_**S2**_	**inline**	2.93	3.01	1.89	2.38
	**skew**	2.94		1.86	

**Figure 5:**
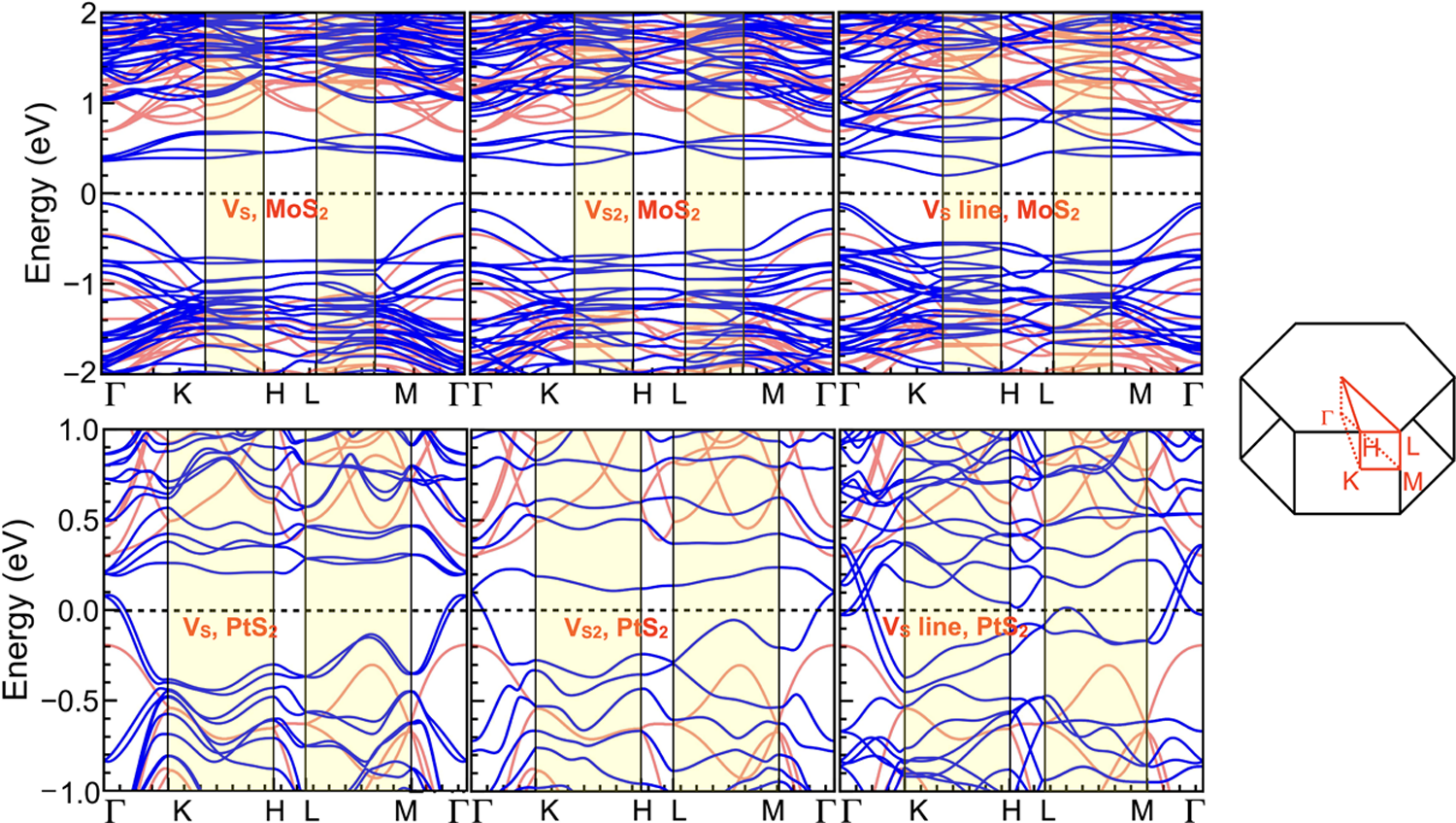
Electronic band structure and Brillouin zone path for *V*_s_ (inline), *V*_S2_ (inline), and *V*_S_ (line defect) for MoS_2_ (top row) and PtS_2_ (bottom row) in solid blue and pristine MoS_2_ and PtS_2_ in semitransparent red. The shaded region in the band diagram corresponds to the out-of-plane directions.

Next, the electronic band structure is analyzed. Note that to correlate the band structure with the transmission spectrum (later calculated), a 3 × 3 bulk supercell is considered. Figure 5 shows the electronic band structure (solid blue) with *V*_S_ (inline), *V*_S2_ (inline), and *V*_S_ (line defect) for MoS_2_ and PtS_2_. The band structures of pristine MoS_2_ and PtS_2_ (in semitransparent red) are plotted in the background. Note that the K–H and L–M correspond to the states in the out-of-plane direction (see the Brillouin zone path aside), of particular interest in this work. In a pristine structure, the Fermi level is far from any states along the out-of-plane direction, suggesting low conductance. For the defect-only structures, although notable variations are observed at the Fermi level for the in-plane wave vectors, there are no significant bands near the Fermi level along the out-of-plane paths. The *V*_S_ (line defect) for PtS_2_, however, shows an exception, where the bands near the Fermi level suggest an increased out-of-plane transport. To further analyze the possibility that the line defect can cause resistivity change due to the application of an external bias, the band structure of different arrangements of the line defect in the bilayer is simulated (S.I. Figure S4). The band structure of such an arrangement shows that in the case where the *V*_S_ concentration is high enough to form an extended line structure, the resultant device will always have a high out-of-plane conductivity irrespective of the relative position of the line defects among different layers. Therefore, although the transport is increased, it is improbable to switch between two appreciably separate conductive states controlled by an external bias. Compared with MoS_2_, *V*_S_ and *V*_S2_ for PtS_2_ also show some bands relatively closer to the Fermi level in the out-of-plane direction. Those bands could affect the out-of-plane transport and the relative effect will be further analyzed making use of quantum transport calculations.

On the other hand for the in-plane direction, it is interesting to note that bands appear much closer to the Fermi level for MoS_2_ and even at the Fermi level for PtS_2_. This shows that the defects could increase the in-plane conductivity, and the ratio of change could be more noticeable for PtS_2_.

#### Metal Intercalation

Following the analysis of the impact of different types of defects and their concentrations on the out-of-plane electronic structure of MoS_2_ and PtS_2_, the role of metallic atoms intercalated in their vdW gaps is studied. The same 3 × 3 × 2 bilayer considered for the study of the vacancy is used here with a single metal atom intercalated in the vdW gap, resulting in a concentration of 1.09 × 10^14^ cm^–2^ for MoS_2_ and 8.7 × 10^13^ cm^–2^ for PtS_2_.

**Figure 6:**
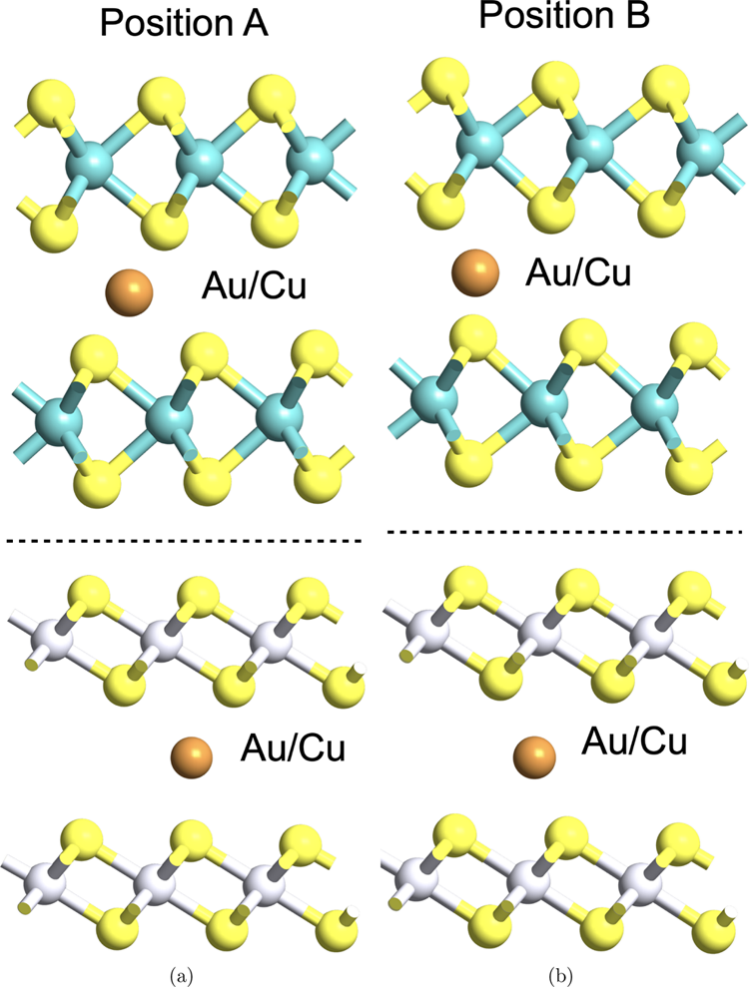
Side view of a 3 × 3 supercell for bilayer MoS_2_ (top row) and PtS_2_ (bottom row) with gold(Au)/copper(Cu) in the vdW gap at (a) position A (left column) and (b) position B (right column).

**Figure 7:**
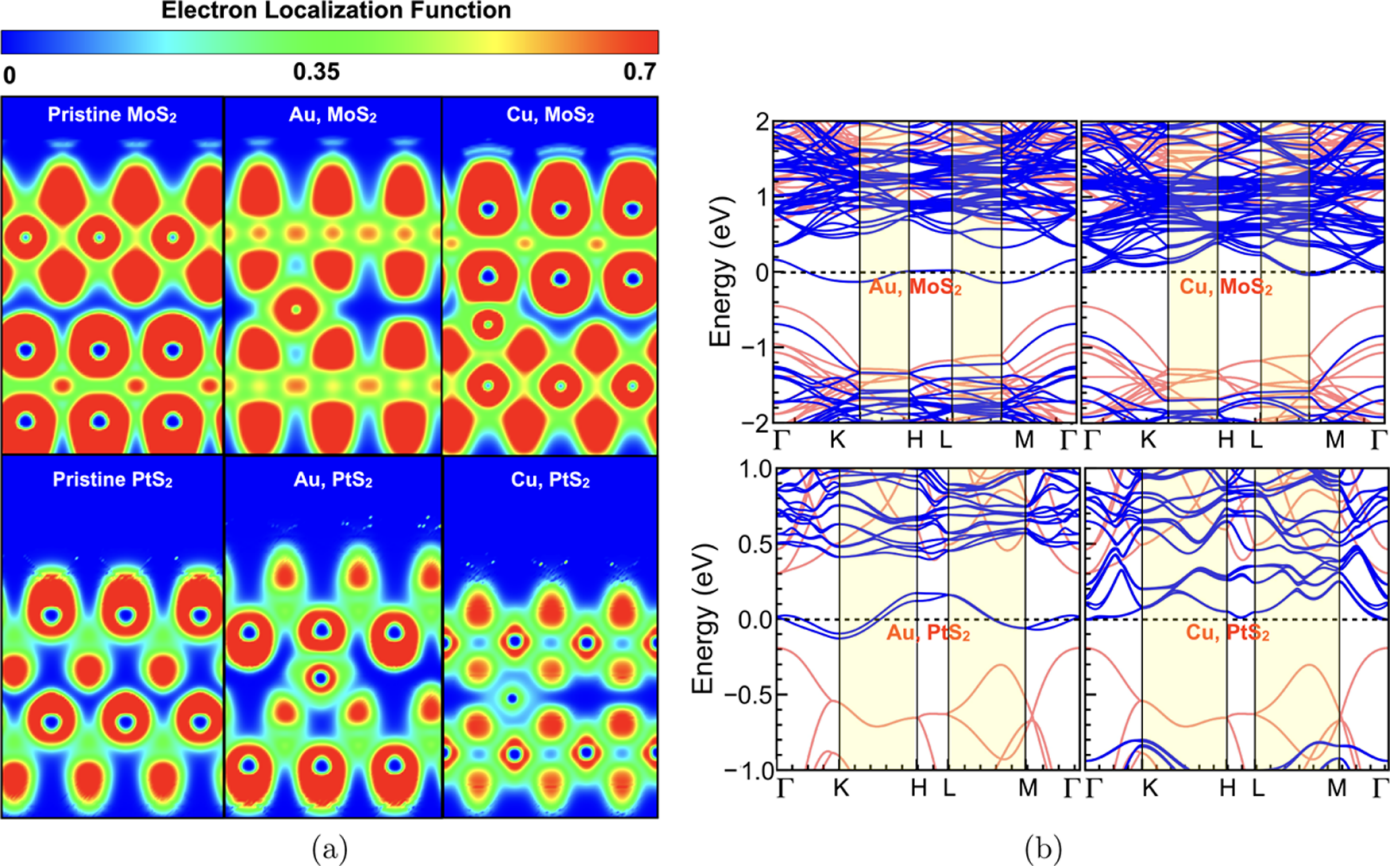
(a) Electron localization function (ELF) and (b) electronic band structure (shown in the solid blue line) for MoS_2_ (top row) and PtS_2_ (bottom row) and for Au (Position A) and Cu (Position B). The band structures for pristine MoS_2_ and PtS_2_ are also shown in semitransparent red. The shaded region in the band diagram corresponds to the out-of-plane directions.

Two different metals are considered: Cu, an active metal with high diffusivity, and Au, a noble metal. Contacts using both metals are related with the generation of memristive behavior in few-layer TMDs.^14,16^ Two different positions of the metal atoms in the vdW gaps, named as A and B in Figure 6, are possible (further details on the exact location in S.I. Figure S5). The *E*_form_ of each configuration is compared in Table 2, from where two conclusions can be drawn: (i) Au is energetically favored in position A, while Cu prefers position B^36^ and (ii) it is energetically less expensive to introduce Cu in the vdW gap than Au. The bilayer distance after the introduction of Au (position A) is 3.61 and 3.92 Å for MoS_2_ and PtS_2_, respectively (Table 2), increasing the vdW gap considerably with respect to the pristine case. However, by examination of the ELF depicted in Figure 7a, it can be seen that the introduction of Au still creates an interlayer bridge, which provides a highly conductive path for electron flow. On the other hand, the bilayer distance on the introduction of Cu (position B) is 3.13 and 2.95 Å for MoS_2_ and PtS_2_, respectively (Table 2), which is significantly smaller than for Au. Note that the bilayer distance is calculated at the intercalation site and therefore gives a localized value of *d*_S–S_. Interestingly, this results in a *d*_S–S_ for the case of Cu-intercalated MoS_2_, smaller than the value in the pristine case, while away from Cu, it is found to be the same as in the pristine MoS_2_. This further suggests the formation of an interlayer bridge, also evident from the ELF in Figure 7a. Furthermore, Cu shows a lower electron probability in the vdW gap of PtS_2_ compared to that of MoS_2_.

**Table 2:** Formation Energy (*E*_form_) and Bilayer Distance (*d*_s–s_) for MoS_2_ and PtS_2_ with Au/Cu in the vdW Gap of the Bilayer

		MoS_2_		PtS_2_	
position	metal	*E*_form_ (eV)	*d*_s-s_ (Å)	*E*_form_ (eV)	*d*_s-s_ (Å)
**A**	**Au**	1.65	3.61	1.84	3.92
	**Cu**	1.52		0.58	
**B**	**Au**	1.83		1.88	
	**Cu**	0.41	3.13	0.51	2.95

Figure 7b shows the electronic band structures for Au (Position A) and Cu (Position B) intercalated in the vdW gap of MoS_2_ and PtS_2_, respectively. One can observe energy states appearing at the Fermi level in the out-of-plane directions, indicating an increase in the conductivity with the introduction of Au/Cu. Correlating the band structure with the ELF, since there seems to be significant delocalization of the electrons in the vdW gap with the introduction of Au, metallic-like states appear at the Fermi level. However, in the case of Cu especially in PtS_2_, although additional states are also observed in the vdW gaps, they are a bit farther from the Fermi level in the out-of-plane direction. Nevertheless, the presence of states very close to the Fermi level could still result in a meaningful transport variation.

### Transport Properties

The transport properties of the system depend on the dispersion of the bands near the Fermi level, as well as the density of the states. Therefore, to get a complete picture of the modulation of the conductivity, the out-of-plane transmission spectrum is computed. The transmission spectrum is calculated for zero bias within the NEGF formalism that makes use of the DFT band structure.

**Figure 8:**
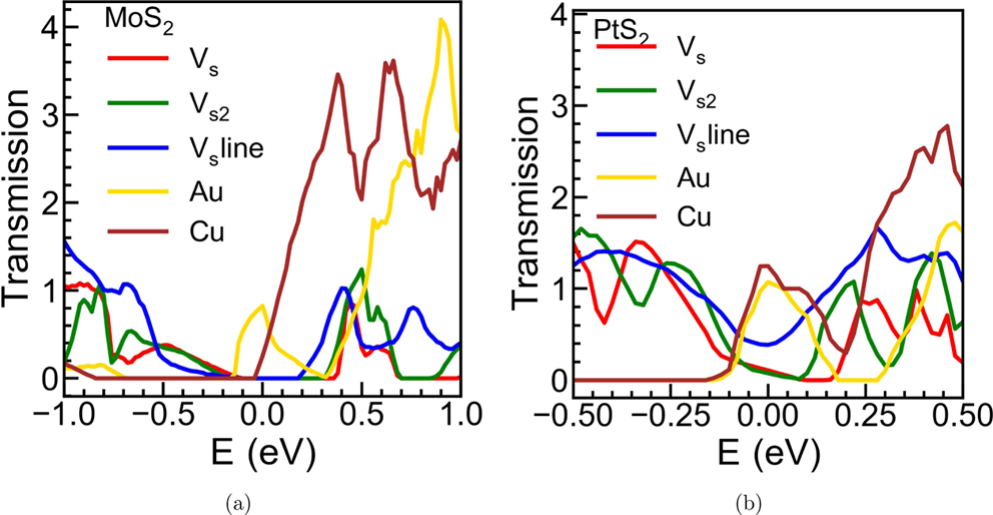
Zero-bias NEGF transmission spectrum comparing *V*_S_ (inline, red), *V*_S2_ (inline, green), *V*_S_ (line defect, blue), Au (Position A), and Cu (Position B) for (a) MoS_2_ and (b) PtS_2_.

Figure 8 compares the transmission spectrum for various configurations of MoS_2_ and PtS_2_. For MoS_2_, the *V*_S_ and *V*_S2_ show zero transmission at the Fermi level, while Au shows a definitive transmission peak and Cu shows a nonzero transmission at the Fermi level in accordance to the band structure (Figure 7b) and ELF (Figure 7a). For PtS_2_, the *V*_S_ and *V*_S2_ show a nonzero transmission at the Fermi level, which is expected due to the presence of bands closer to the Fermi level in the out-of-plane direction. However, compared to the structure with Au or Cu, the transmission value is significantly small. Both Au and Cu give a definitive transmission peak at the Fermi level, suggesting a modulation of the conductivity. Note that the presence of metallic atoms in the vdW gap can be controlled by an external bias or temperature, producing the device switching between the observed high and low conductive states. On the contrary, the *V*_S_ line defect for PtS_2_, despite showing a significant nonzero transmission at the zero Fermi level, cannot give rise to external stimuli-dependent resistive switching as discussed earlier.

## Discussion

The change in the out-of-plane conductivity, and the consequent resistive switching, has been investigated in this work for two representative members of the TMD family in the presence of intrinsic point defects (sulfur vacancies) and external metallic atoms using DFT and NEGF calculations.

In particular, the arrangement of sulfur vacancies in the MoS_2_ and PtS_2_ crystals has been examined based on their *E*_form_. It was also found that *V*_S_ tends to form extended line defects, in agreement with the experiments. The formation energy of vacancies in the bilayer structure has also been investigated when *V*_S_ and *V*_S2_ are vertically aligned or skewed, remaining almost unaltered. This reveals that the presence of a vacancy in one of the layers, in principle, does not affect the position of the vacancy in the adjacent layer. The vdW bilayer distance for pristine MoS_2_ and PtS_2_, however, reduces when either *V*_S_ or *V*_S2_ was introduced, thereby decreasing the tunneling barrier width. Despite this, the ELF plots have not shown signs of an interlayer physical bridge, suggesting a low out-of-plane conductivity mediated by vacancies, contrary to the presence of the metal atoms in the vdW gap, that evidenced the formation of interlayer bridges. The exception to this result was Cu in PtS_2_, where the probability of finding electrons in the vdW gap was relatively low.

The band structure analysis demonstrated that the presence of defects introduces states along the in-plane directions, as previous investigations corroborate.^19^ However, they do not originate any significant dispersion bands near the Fermi level in the out-of-plane direction. An exception to this situation was the *V*_S_ line defect in PtS_2_, where states appear at the Fermi level in the out-of-plane direction. However, as such defects force the structure to be permanently in a highly conductive state, they proved to be ineffective in achieving resistance switching controlled by the external bias. Introduction of the atoms of Au/Cu in the vdW gap resulted in metallic-like states at the Fermi level in the out-of-plane wave vector path, originating an increased out-of-plane conductivity. The Cu in PtS_2_, which showed low electron probability in the vdW gap, did not create metallic-like states but created states very close to the Fermi level in the out-of-plane direction, which could still considerably affect the conductivity.

To further corroborate the change in the conductivity of the above structures, NEGF calculations were carried out to estimate the transmission spectrum in the out-of-plane direction. For MoS_2_, the different defect configurations evaluated did not show transmission at the Fermi level, whereas Au and Cu show increased transmission probability along the out-of-plane direction close to the Fermi level energy. For PtS_2_, the *V*_S_ and *V*_S2_ defect configurations had negligible transmission at the Fermi level compared to Au/Cu that presented a relatively higher transmission value. According to the band structure, the *V*_S_ line defect also showed a nonzero transmission in the out-of-plane direction.

## Conclusions

To conclude, the intrinsic sulfur vacancy defect usually present in the semiconducting TMDs, MoS_2_ and PtS_2_, appears to be insufficient to cause noticeable conductivity changes on application of an external bias and therefore is inefficient for triggering the desired resistive switching. On the contrary, the presence of metallic atoms in the vdW gap demonstrated to be of the utmost importance to trigger the memristive process, emphasizing how the proper choice of the metal electrode is critical in the fabrication and optimization of multilayer TMD-based memristors. Since both MoS_2_ and PtS_2_ are representative of the crystal structures of a wide range of TMDs, the results obtained here could, in principle, be extended to other semiconducting TMDs. For the completeness of the discussion, it is also worthwhile to note that other extended forms of defects such as vacancy clusters (MoS_3_, MoS_6_, etc.), grain boundaries, or dislocations are also routinely present in the TMDs. However, the analysis in this work is limited to single-crystalline TMDs and the most commonly found defects. The impact of grain boundaries on out-of-plane transport and their precise manipulation using an external bias remain an active area of study beyond the scope of the present work.

## Supporting Information Available

Verification of the DFT simulation setup and details on defect formation energy and various structural configurations (PDF)
