# Supplementary material for: Role of Point Defects and Ion Intercalation in Two-Dimensional Multilayer Transition Metal Dichalcogenide Memristors
Source: ACS Appl Nano Mater. 2024 Oct 24;7(21):24857–65. doi: 10.1021/acsanm.4c04769 (PMC11558805; doi:10.1021/acsanm.4c04769)
Supplement: Supplementary file 1 — an4c04769_si_001.pdf [file an4c04769_si_001.pdf]

Supporting Information

Role of Point Defects and Ion Intercalation in Two-Dimensional Multilayer Transition Metal Dichalcogenide Memristors

Mohit D. Ganeriwala,[*,†] Alejandro Toral-López,[‡] Estela Calaforra-Ayuso,[†] Francisco Pasadas,[†] Francisco G. Ruiz,[†] Enrique G. Marin,[†] and Andres Godoy[*,†]

†*Department of Electronics and Computer Technology, University of Granada, 18071, Granada, Spain*

‡*University of Pisa, Italy*

E-mail: mohit@go.ugr.es; agodoy@ugr.es

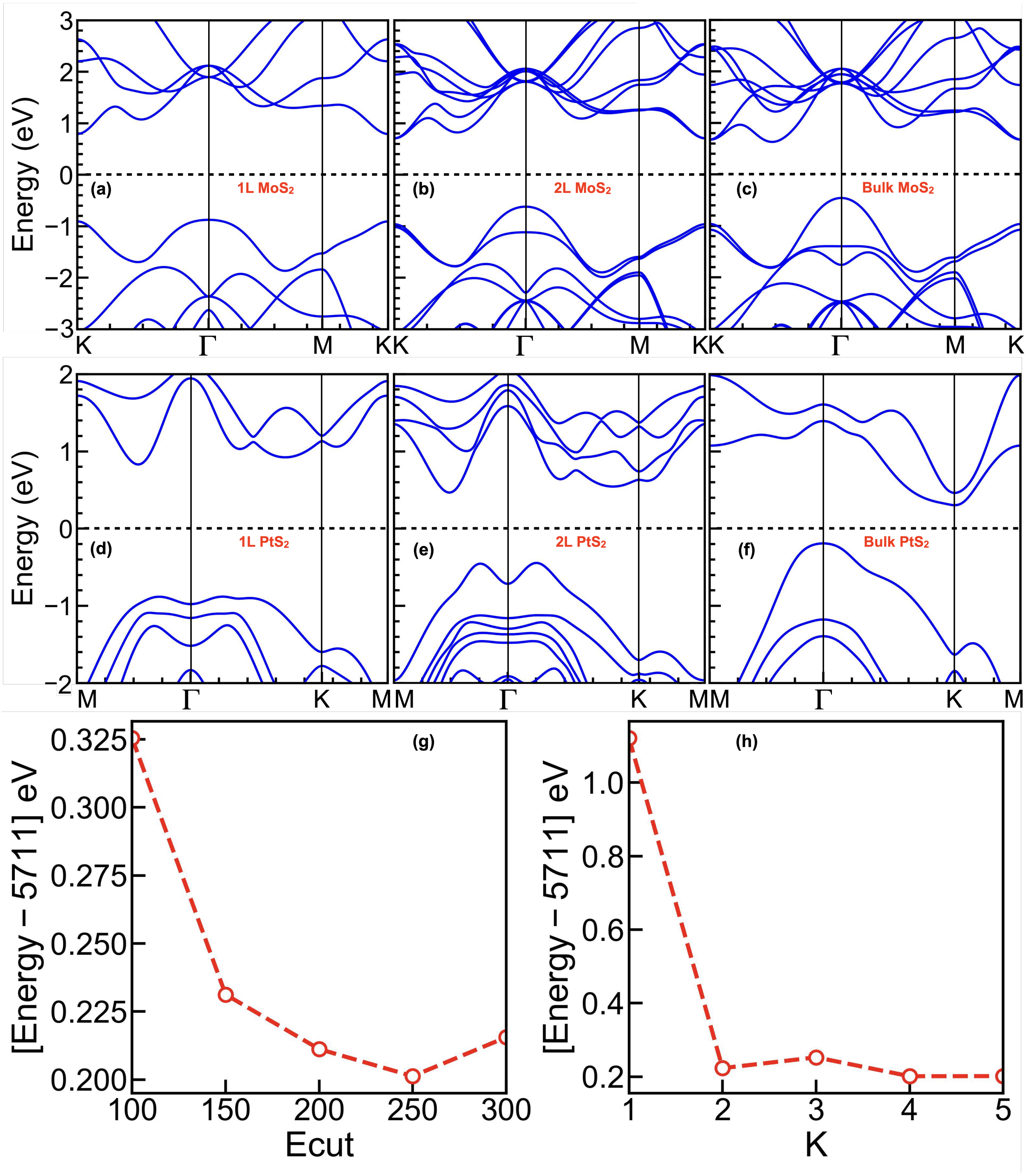

Figure S1: Electronic band structure of (a) monolayer, (b) bi-layer, (c) bulk, MoS_2 (top row), (d) monolayer, (e) bi-layer and (f) bulk, PtS_2 (bottom row) and DFT energy minimization as a function of (g) Cutt-off Energy (Ecut) for k point density (Å) of 4x4x4 and (h) K (k-point density = KxKxK) for Ecut of 250 Ry for bulk MoS_2, showing the rational for choosing Ecut of 250 Ry and K point density of 4x4x4. The band structure agrees well with those reported in literature.[1,2]

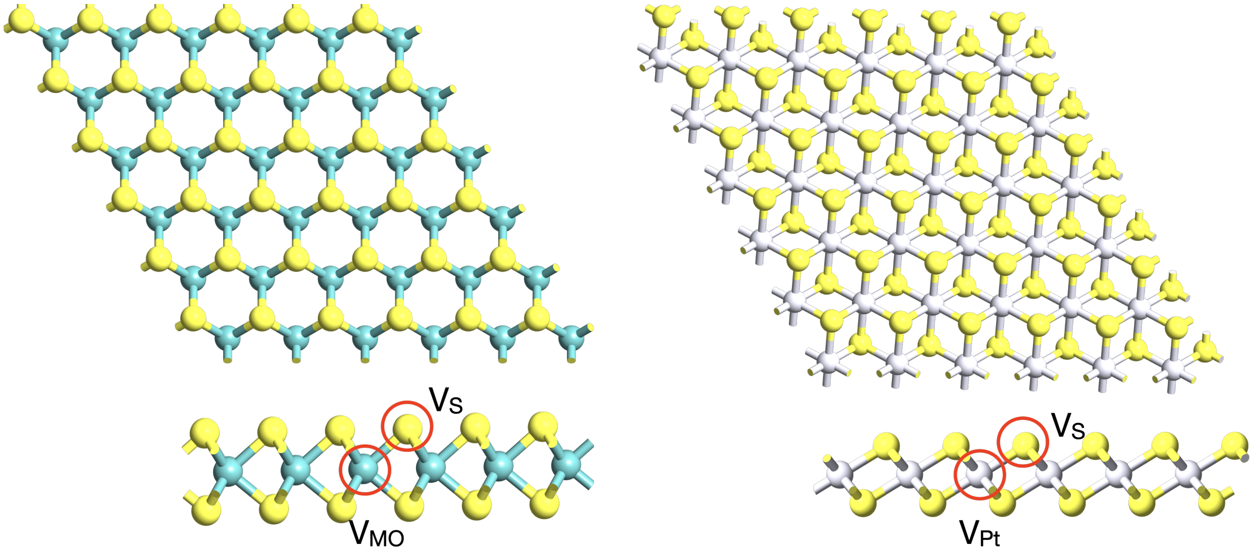

(a)

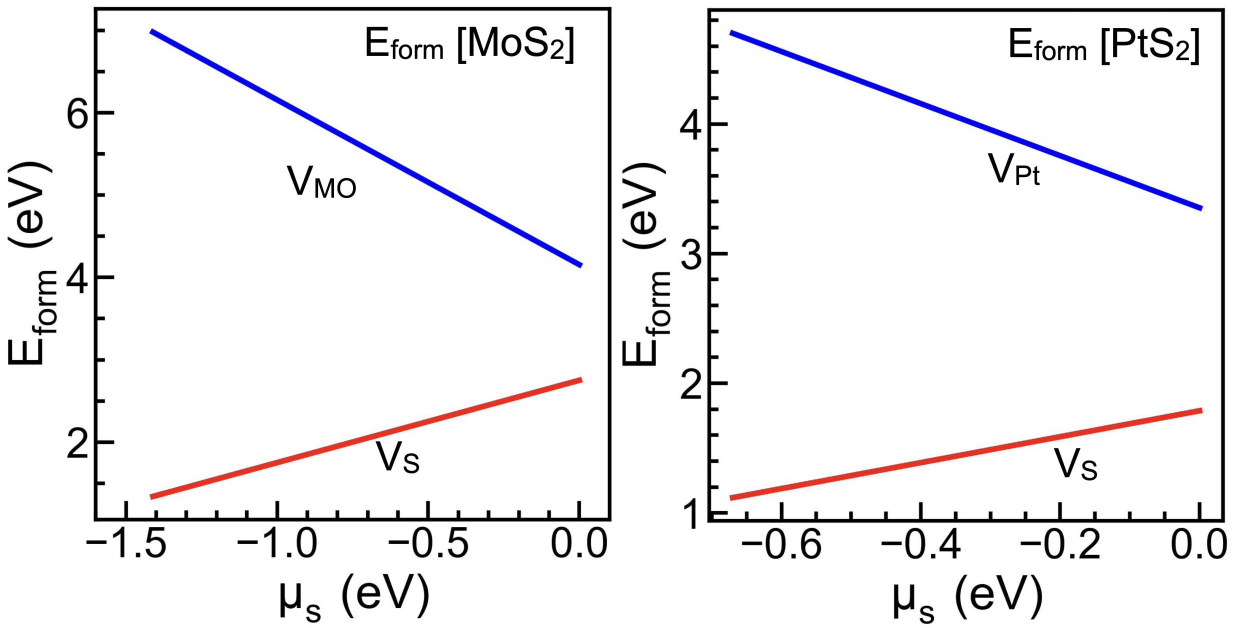

(b)

Figure S2: (a) Top and side view of a 6×6 supercell with single sulfur point defect (V_s) and single Mo and Pt vacancy defect (V_{MO} and V_{Pt}) and (b) formation energy (E_{form}) as a function of sulfur chemical potential (μ_S), for V_s, V_{MO} and V_{Pt} for MoS_2 and PtS_2.

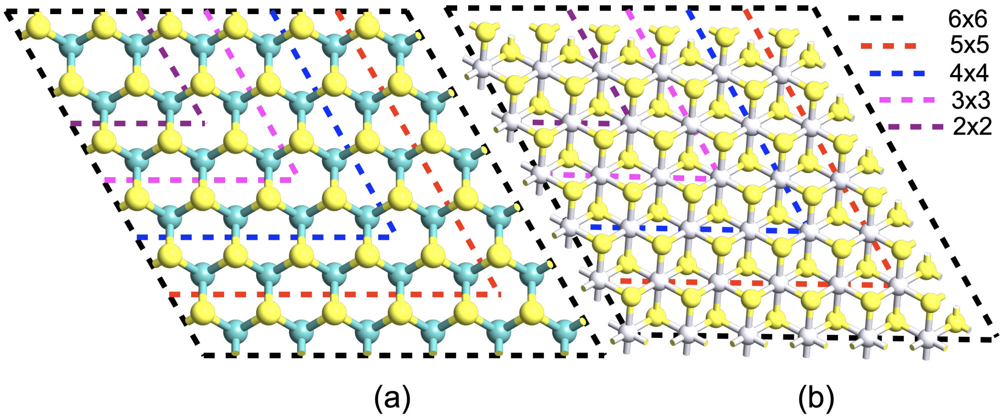

Figure S3: Supercell of (a) MoS_2 and (b) PtS_2 showing different sizes ranging from 6×6 to 2×2.

Table S1: Formation energy (E_{form}) of single sulfur vacancy (V_S) point defect for MoS_2 and PtS_2 with varying supercell size.

**Supercell size**	$\mathbf{E_{form}[MoS_2]}$ **(eV)**	$\mathbf{E_{form}[PtS_2]}$ **(eV)**
**6x6**	2.75	1.79
**5x5**	2.75	1.79
**4x4**	2.75	1.79
**3x3**	2.75	1.79
**2x2**	2.84	1.84

Table S2: Formation energies (E_{form}) of a bilayer MoS_2 and PtS_2 for defects in the top layer calculated with reference to a V_S in the bottom layers. This suggest that given a V_S in the bottom layer the next defect generated in the top layer is also preferentially V_S.

$\mathbf{MoS_2}$		$\mathbf{PtS_2}$	
**Defect Type**	**Formation Energy (eV)**	**Defect Type**	**Formation Energy (eV)**
Sulfur Vacancy (V_S)	2.99	Sulfur Vacancy (V_S)	1.83
Molybdenum Vacancy (V_{Mo})	4.72	Platinum Vacancy (V_{Pt})	2.83

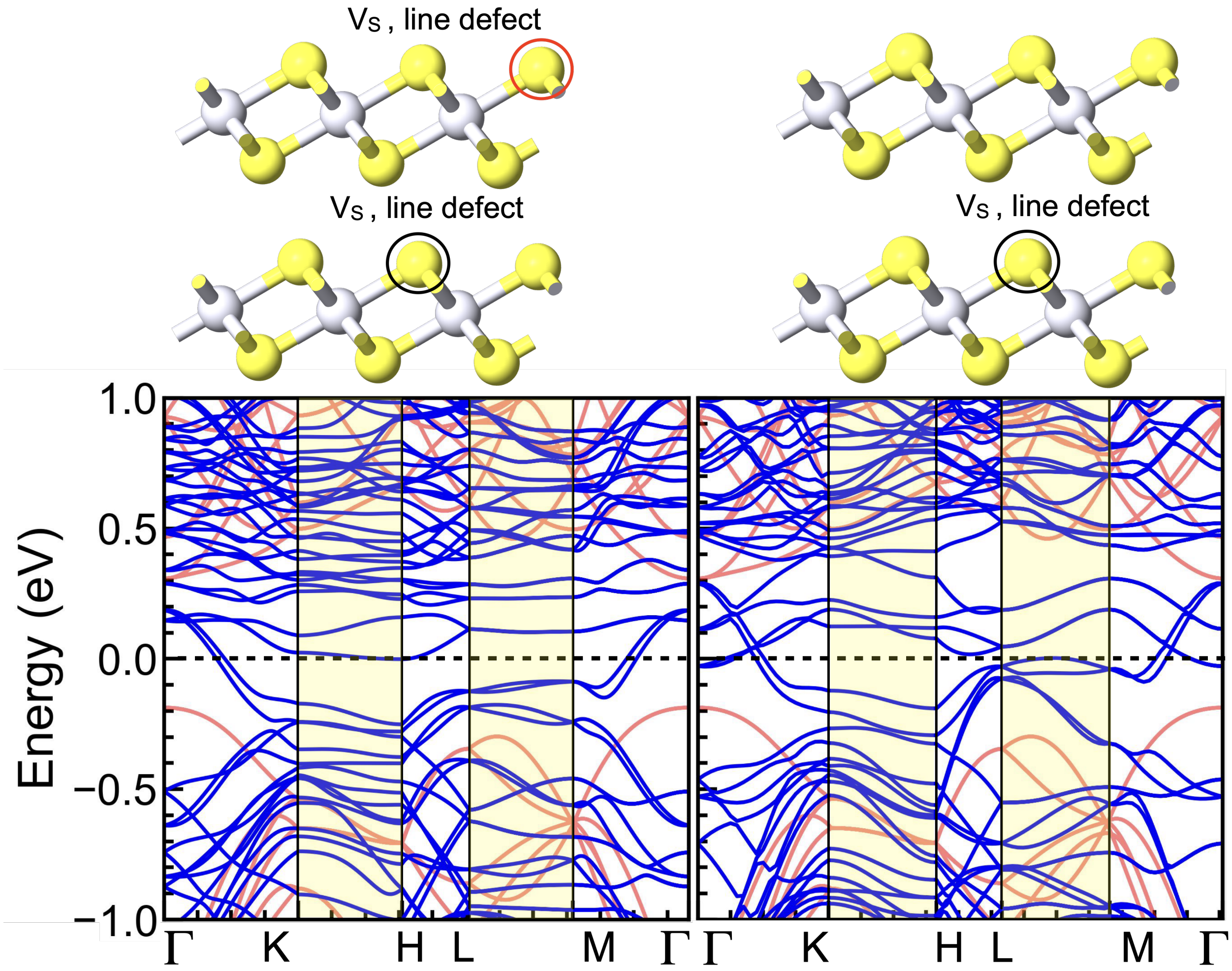

Figure S4: Supercell including defect and electronic band structure of bulk PtS_2, with V_S, line defect (defect-line going perpendicular to the paper) skewed between two layers and in only one layer, with adjacent layer being pristine. The shaded regions in the band diagram corresponds to the out-of-plane directions.

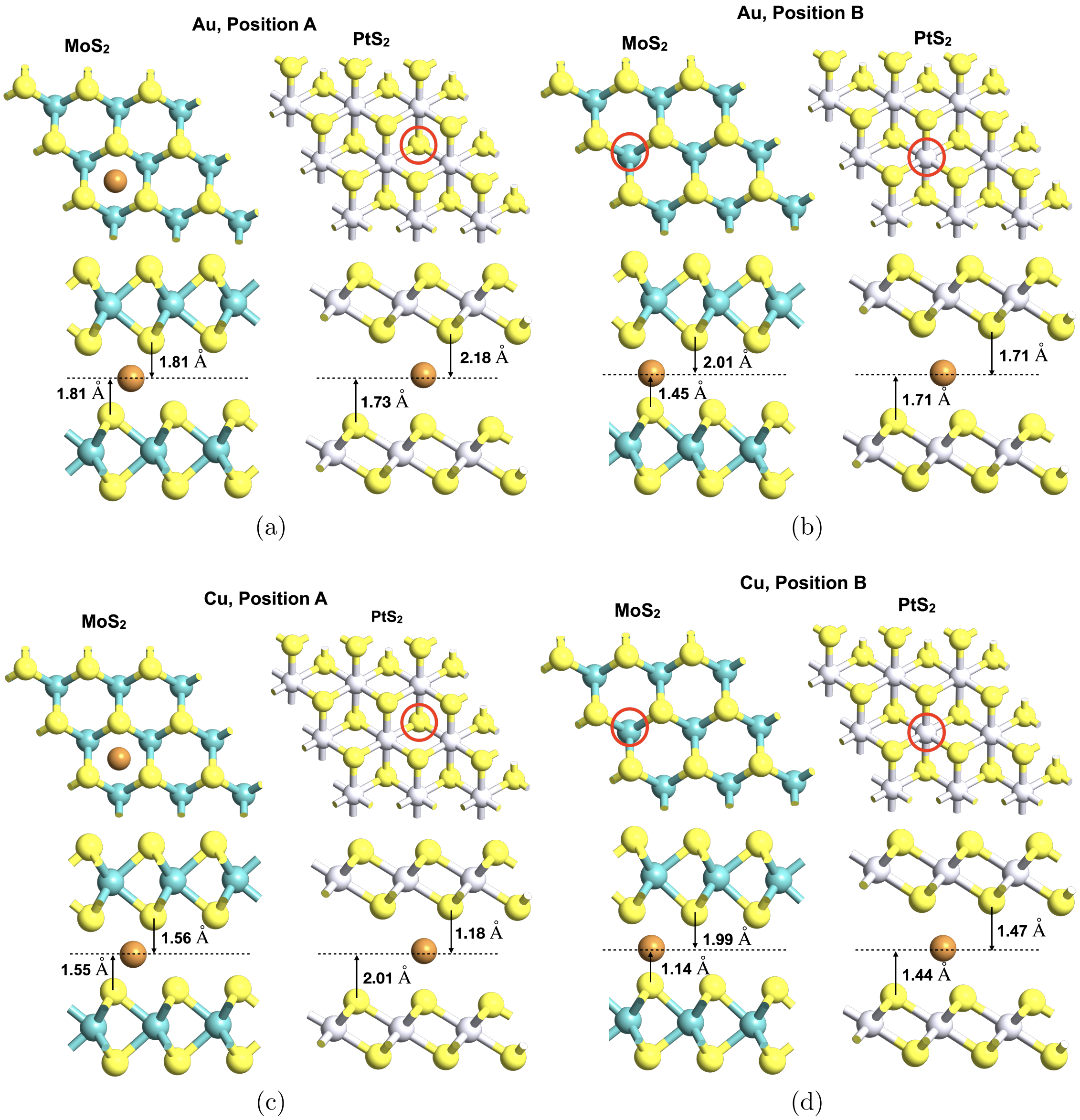

Figure S5: Top and side view of MoS_2 and PtS_2 bilayer with intercalated (a) Au, at Position A, (b) Au, Position B, (c) Cu, Position A and (d) Cu, Position B, along with their respective distance from both layers.

References

(1) Lu, A. K. A.; Pourtois, G.; Luisier, M.; Radu, I. P.; Houssa, M. On the electrostatic control achieved in transistors based on multilayered MoS_2: A first-principles study. *Journal of Applied Physics* **2017**, *121*.

(2) Villaos, R. A. B.; Crisostomo, C. P.; Huang, Z.-Q.; Huang, S.-M.; Padama, A. A. B.; Albao, M. A.; Lin, H.; Chuang, F.-C. Thickness dependent electronic properties of Pt dichalcogenides. *npj 2D Materials and Applications* **2019**, *3*, 2.
